# ITGB1-DT Facilitates Lung Adenocarcinoma Progression via Forming a Positive Feedback Loop With ITGB1/Wnt/β-Catenin/MYC

**DOI:** 10.3389/fcell.2021.631259

**Published:** 2021-03-04

**Authors:** Ruimin Chang, Xiaoxiong Xiao, Yao Fu, Chunfang Zhang, Xiaoyan Zhu, Yang Gao

**Affiliations:** ^1^Department of Thoracic Surgery, Xiangya Hospital, Central South University, Changsha, China; ^2^Hunan Engineering Research Center for Pulmonary Nodules Precise Diagnosis & Treatment, Changsha, China; ^3^National Clinical Research Center for Geriatric Disorders, Changsha, China; ^4^Hunan Key Laboratory of Skin Cancer and Psoriasis, Changsha, China; ^5^Department of Anesthesiology, Xiangya Hospital, Central South University, Changsha, China

**Keywords:** ITGB1-DT, lung adenocarcinoma, progression, feedback loop, integrin β1, Wnt/β-catenin pathway, MYC

## Abstract

Lung adenocarcinoma (LUAD) is the main histological type of lung cancer, which is the leading cause of cancer-related deaths. Long non-coding RNAs (lncRNAs) were recently revealed to be involved in various cancers. However, the clinical relevance and potential biological roles of most lncRNAs in LUAD remain unclear. Here, we identified a prognosis-related lncRNA ITGB1-DT in LUAD. ITGB1-DT was upregulated in LUAD and high expression of ITGB1-DT was correlated with advanced clinical stages and poor overall survival and disease-free survival. Enhanced expression of ITGB1-DT facilitated LUAD cellular proliferation, migration, and invasion, and also lung metastasis *in vivo*. Knockdown of ITGB1-DT repressed LUAD cellular proliferation, migration, and invasion. ITGB1-DT interacted with EZH2, repressed the binding of EZH2 to *ITGB1* promoter, reduced H3K27me3 levels at *ITGB1* promoter region, and therefore activated *ITGB1* expression. Through upregulating ITGB1, ITGB1-DT activated Wnt/β-catenin pathway and its downstream target MYC in LUAD. The expressions of ITGB1-DT, ITGB1, and MYC were positively correlated with each other in LUAD tissues. Intriguingly, ITGB1-DT was found as a transcriptional target of MYC. MYC directly transcriptionally activated ITGB1-DT expression. Thus, ITGB1-DT formed a positive feedback loop with ITGB1/Wnt/β-catenin/MYC. The oncogenic roles of ITGB1-DT were reversed by depletion of ITGB1 or inhibition of Wnt/β-catenin pathway. In summary, these findings revealed ITGB1-DT as a prognosis-related and oncogenic lncRNA in LUAD via activating the ITGB1-DT/ITGB1/Wnt/β-catenin/MYC positive feedback loop. These results implicated ITGB1-DT as a potential prognostic biomarker and therapeutic target for LUAD.

## Introduction

Lung cancer is still the most frequently diagnosed malignancy and the leading cause of mortality among all malignancies, accounting for 11.6% of the total cancer cases and 18.4% of the total cancer deaths worldwide (Bray et al., 2018). The histological types of lung cancer include lung adenocarcinoma (LUAD), lung squamous cell carcinoma (LUSC), large cell lung cancer, and small cell lung cancer. LUAD is the most common type, which accounts for 40%–50% of all lung cancers (Liang et al., 2020; Cooke et al., 2020). Although most early-stage LUAD could be effectively treated by surgical resection, the prognoses of unresectable LUAD are still very poor with 5-year survival rates less than 20% (Devarakonda et al., 2020). Recent advances in chemotherapy, molecule targeted therapy, and immunotherapy show efficient restriction of LUAD progression. However, almost all LUAD patients lastly emerge resistance (Garon et al., 2015). Therefore, further investigation of the molecular mechanisms underlying LUAD development is essential for developing novel treatment against LUAD.

The identification of critical genes involved in LUAD provided several therapeutic intervention targets, such as EGFR, ALK, HER2, MET, VEGF, and PD-1 (Cataldo et al., 2011; Marjanovic et al., 2020). Apart from these protein-encoding genes, increasing evidences have revealed the extensive implications of non-coding RNAs in multiple physiological and pathological steps (Ernst et al., 2018; Esposito et al., 2019; Hanniford et al., 2020; Daneshvar et al., 2020; Li Y. et al., 2020; Budkova et al., 2020). Among these non-coding RNAs, microRNAs have been sufficiently demonstrated by us and others to exert critical regulatory roles in various cancers (Yuan et al., 2011; Chang et al., 2014, 2017). Long non-coding RNAs (lncRNAs) are another class of non-coding RNAs with more than 200 nucleotides in length (Ponting et al., 2009; Zhang Y. et al., 2020). lncRNAs have been gradually revealed to exhibit regulatory roles in a variety of cancers (Schmitt and Chang, 2016). Multiple cancer-related lncRNAs have been identified, such as MALAT1, HOTAIR, lncRNA-ATB, FAL1, EPIC1, LINK-A, and PXN-AS1 (Gutschner et al., 2013; Hu et al., 2014; Yuan et al., 2014; Lin et al., 2016; Yuan et al., 2017; Wang et al., 2018; Li H. et al., 2020). In lung cancer, lncRNAs PIK3CD-AS2, HCP5, SNHG15, SNHG11, CASC9, and ALAL-1 have oncogenic roles (Athie et al., 2020; Cheng et al., 2020; Chen Z. et al., 2020; Huang et al., 2020; Li C. et al., 2020; Zheng et al., 2020). In contrast, lncRNAs NORAD, BRCAT54, CASC7, and HSPC324 have tumor-suppressive roles (Tan et al., 2019; Chen L. et al., 2020; Jafarzadeh et al., 2020; Yang et al., 2020). High-throughput transcriptome sequencings have found more than 58,000 lncRNAs in human cells (Iyer et al., 2015). Thus, only a small part of these numerous lncRNAs have been studied in cancers. Whether other lncRNAs are involved in cancers, particularly in LUAD, needs further investigation.

To search potential lncRNAs associated with LUAD progression, we analyzed The Cancer Genome Atlas (TCGA) LUAD dataset and identified ITGB1-DT as an upregulated and poor-prognosis correlated lncRNA in LUAD. *ITGB1-DT* is located on chromosome 10p11.22 and is transcribed in the opposite direction from coding gene *ITGB1*. *ITGB1-DT* has four exons and encodes transcript (ITGB1-DT) with 1078 nucleotides. ITGB1-DT was previously reported to be related to poor prognosis of clear cell renal cell carcinoma and gastric cancer (Han et al., 2017; Jiang et al., 2019). However, the expression, roles, and clinical relevance of ITGB1-DT in LUAD are unknown. In this study, we further investigated the clinical relevance, roles, and mechanisms of action of ITGB1-DT in LUAD.

## Materials and Methods

### Clinical Samples

A total of 64 pairs of LUAD tissues and matched normal lung tissues were randomly selected from LUAD patients who received lung lobectomy at Xiangya Hospital. All clinical samples were confirmed by histopathological examination. We obtained written informed consents from all participants. The study protocol was approved by the Ethics Committee of Xiangya Hospital. We conducted this study in accordance with the ethical standards of our hospital and the Helsinki Declaration. The clinicopathological features of these 64 LUAD samples are summarized in Table 1.

**TABLE 1:** Correlations of ITGB1-DT with clinicopathological features of LUAD.

**Clinicopathologic Variable**	***n***	**ITGB1-DT**		***P* value**
		**Low**	**High**	
Gender				0.802
Male	33	17	16	
Female	31	15	16	
Age				0.434
≥ 60	41	22	19	
< 60	23	10	13	
Tumor size				0.424
> 5 cm	21	9	12	
≤ 5 cm	43	23	20	
Local invasion				0.048
T1–2	47	27	20	
T3–4	17	5	12	
Lymphatic metastasis				0.024
N0	35	22	13	
N1–3	29	10	19	
TNM stage				0.017
I–II	43	26	17	
III–IV	21	6	15	

### Cell Culture and Treatment

Human bronchial epithelial cell line 16HBE was purchased from Millipore (Bedford, MA, United States). Human LUAD cell lines A549 (ATCC CCL-185), NCI-H1975 (ATCC CRL-5908), and NCI-H1299 (ATCC CRL-5803) were acquired from the American Type Culture Collection (Manassas, VA, United States). 16HBE was cultured in Airway Epithelial Cell Basal Media (ATCC) supplemented with Bronchial/Tracheal Epithelial Cell Growth Kit components (ATCC). A549 was cultured in F-12K Medium (Invitrogen, Carlsbad, CA, United States) added with 10% fetal bovine serum (FBS, Invitrogen). NCI-H1975 and NCI-H1299 cells were cultured in RPMI-1640 Medium (Invitrogen) added with 10% FBS. These cells were cultured in 5% CO_2_ at 37°C. Where indicated, LUAD cells were treated with 200 ng/ml Wnt3a (R&D Systems, Minneapolis, MN, United States) or 25 μM ICG-001 (Selleck, Houston, TX, United States) for the indicated time.

### Quantitative Real-Time PCR (qRT-PCR)

Total RNA was extracted from indicated cells or frozen specimens using Trizol reagents (Invitrogen). Reverse transcription was performed using M-MLV Reverse Transcriptase (Invitrogen) and the extracted RNA. qRT-PCR was conducted on the Applied Biosystems 7300 Fast Real-Time PCR System (Applied Biosystems, Foster City, CA, United States) using SYBR^®^ Green Realtime PCR Master Mix (Toyobo, Osaka, Japan) and the primers 5′-TGTATGTGATGGCTTTGGAG-3′ (forward) and 5′-AGGTGGGCAGTGGTATGG-3′ (reverse) for ITGB1-DT, 5′-GTTTGTAGGAAGAGGGATAA-3′ (forward) and 5′-CCA GTGTAGTTGGGGTTG-3′ (reverse) for ITGB1, 5′-GTCGG AGTCAACGGATTTG-3′ (forward) and 5′-TGGGTGGAATC ATATTGGAA-3′ (reverse) for GAPDH, 5′-GGAGCAGAA AACAGCAGG-3′ (forward) and 5′-GGAAGGGGTCAGGAG AAA-3′ (reverse) for MALAT1, 5′-CTTCCCCTACCCTCTCAA-3′ (forward) and 5′-CGATTTCTTCCTCATCTTCT-3′ (reverse) for MYC, 5′-GAACGGATTTAGAAGCCTG-3′ (forward) and 5′-GGTCCTCCATTTCAGCCT-3′ (reverse) for HOTAIR, 5′-G CCCCATCAGGCCTCCGTTT-3′ (forward) and 5′-ACCT TGCCTTCTTTGTCTTTGTTGGA-3′ (reverse) for E-cadherin, 5′-TGGACCATCACTCGGCTTA-3′ (forward) and 5′-ACA CTGGCAAACCTTCACG-3′ (reverse) for N-cadherin, and 5′-CCTGAACCTGAGGGAAACTAA-3′ (forward) and 5′-GCA GAAAGGCACTTGAAAGC-3′ (reverse) for Vimentin (Yuan et al., 2014). The quantification of ITGB1-DT, ITGB1, and MYC expressions was normalized to GAPDH. The results were analyzed using the 2^−ΔΔCt^ method.

### Vector Construction, siRNAs, and Transfection

ITGB1-DT full-length sequence was PCR-amplified using the PrimeSTAR^®^ Max DNA Polymerase (Takara, Shiga, Japan) and the primers 5′-CCCAAGCTTGCTTTTCTGGAGTGATAA TG-3′ (forward) and 5′-GCTCTAGAGTAATCAAATAAAACTT ACAGTGGT-3′ (reverse). After digestion by the restriction enzymes *Hin*dIII (Takara) and *Xba*I (Takara), the PCR products were inserted into pcDNA3.1 vector (Invitrogen) and pSPT19 vector (Roche, Mannheim, Germany) to generate ITGB1-DT overexpression vector pcDNA3.1-ITGB1-DT and ITGB1-DT *in vitro* transcription vector pSPT19-ITGB1-DT, respectively. Coding sequence (CDS) of MYC was PCR-amplified using the PrimeSTAR^®^ Max DNA Polymerase (Takara) and the primers 5′-CCCAAGCTTGCCAGGACCCGCTTCT-3′ (forward) and 5′-G CTCTAGAGGTGATTGCTCAGGACATTTC-3′ (reverse). After digestion by the restriction enzymes *Hin*dIII and *Xba*I, the PCR products were inserted into pcDNA3.1 vector (Invitrogen) to generate MYC overexpression vector pcDNA3.1-MYC. CDS of ITGB1 was PCR-amplified using the PrimeSTAR^®^ Max DNA Polymerase (Takara) and the primers 5′-GGAATTCAGCG GGAGTCGCGGAACA-3′ (forward) and 5′-GCTCTAGAGG ATTTGCACGGGCAGTAC-3′ (reverse). After digestion by the restriction enzymes *Eco*RI and *Xba*I, the PCR products were inserted into pcDNA3.1 vector (Invitrogen) to generate ITGB1 overexpression vector pcDNA3.1-ITGB1. ON-TARGETplus Human MYC siRNA SMART Pool (Cat: L-003282-02-0010), ON-TARGETplus Human ITGB1 siRNA SMART Pool (Cat: L-004506-00-0010), and ON-TARGETplus Human EZH2 siRNA SMART Pool (Cat: L-004218-00-0010) were purchased from Dharmacon (Cambridge, United Kingdom). The transfection and co-transfection of vectors and siRNAs were conducted using Lipofectamine 3000 (Invitrogen).

### Stable Cell Line Construction

To construct LUAD cells with ITGB1-DT stable overexpression, ITGB1-DT overexpression vector pcDNA3.1-ITGB1-DT was transfected into A549 and NCI-H1975 cells. Seventy-two hours later, the transfected cells were treated with neomycin to select ITGB1-DT stably overexpressed LUAD cells. Two pairs of cDNA oligonucleotides targeting ITGB1-DT were synthesized and inserted into the shRNA lentivirus expressing vector pLV6/EF-1aF/Puro (GenePharma, Shanghai, China), which were further used to produce shRNA lentiviruses targeting ITGB1-DT. One pair of cDNA oligonucleotides targeting ITGB1 were synthesized and inserted into pLV6/EF-1aF/Puro, which were further used to produce shRNA lentiviruses targeting ITGB1. Scrambled non-targeting cDNA oligonucleotides were used as negative control (NC). The cDNA oligonucleotides sequences were as follows: for shRNA-ITGB1-DT-1, 5′-GATCCGGAGCAAATTGATT GACAATGTTCAAGAGACATTGTCAATCAATTTGCTCCTT TTTTG-3′ (forward) and 5′-AATTCAAAAAAGGAGCAAAT TGATTGACAATGTCTCTTGAACATTGTCAATCAATTTGC TCCG-3′ (reverse); for shRNA-ITGB1-DT-2, 5′-GATCCGGTC TAGCTGAGTTGACAAGATTCAAGAGATCTTGTCAACTCA GCTAGACCTTTTTTG-3′ (forward) and 5′-AATTCAAAA AAGGTCTAGCTGAGTTGACAAGATCTCTTGAATCTTGTC AACTCAGCTAGACCG-3′ (reverse); for shRNA-ITGB1, 5′-GATCCGGATACTAGTACTTGTGAAGCTTCAAGAGAGCTT CACAAGTACTAGTATCCTTTTTTG-3′ (forward) and 5′-AA TTCAAAAAAGGATACTAGTACTTGTGAAGCTCTCTTGAA GCTTCACAAGTACTAGTATCCG-3′ (reverse); for shRNA-NC, 5′-GATCCGTTCTCCGAACGTGTCACGTTTCAAGAGAACG TGACACGTTCGGAGAACTTTTTTG-3′ (forward) and 5′-AATTCAAAAAAGTTCTCCGAACGTGTCACGTTCTCTTGA AACGTGACACGTTCGGAGAACG-3′ (reverse). To construct LUAD cells with ITGB1-DT stable silencing, the shRNA lentiviruses targeting ITGB1-DT were infected into A549 and NCI-H1299 cells. Ninety-six hours later, the infected cells were treated with puromycin to select ITGB1-DT stably silenced LUAD cells. To construct LUAD cells with ITGB1-DT stable overexpression and concurrent ITGB1 stable silencing, the shRNA lentiviruses targeting ITGB1 were infected into A549 cells with ITGB1-DT stable overexpression. Ninety-six hours later, the infected cells were treated with puromycin and neomycin to select ITGB1-DT stably overexpressed and ITGB1 stably silenced A549 cells.

### Cell Proliferation Assay

Cell proliferation abilities were evaluated using Cell counting kit-8 (CCK-8) and 5-ethynyl-2′-deoxyuridine (EdU) incorporation assays as previously described (Wu et al., 2020). Briefly, for CCK-8 assay, 3000 indicated LUAD cells were plated into 96-well plates per well. After incubation for 24 h, 48 h, or 72 h, 10 μl of CCK-8 solution (Dojindo, Kumamoto, Japan) was added per well. After incubation for another 2 h, the absorbance at 450 nm was measured. EdU incorporation assay was conducted using the EdU Kit (Roche) according to the manufacturer’s manual. The results were quantified by the Zeiss photomicroscope (Carl Zeiss, Oberkochen, Germany) via counting at least five random fields.

### Cell Migration and Invasion Assays

Cell migration and invasion abilities were evaluated using transwell migration and invasion assays as we previously described (Chang et al., 2017). Briefly, 1 × 10^5^ indicated LUAD cells resuspended in FBS-free medium were seeded into the upper chambers of transwell inserts (Millipore). For transwell invasion assay, the inserts were pre-coated with matrigel (1:30 dilution) (BD Biosciences, Franklin Lakes, NJ, United States). For transwell migration assay, the inserts were not pre-coated with matrigel. Medium containing 10% FBS was added into the under chambers. After incubation at 37°C for another 48 h, the cells in the upper chambers were removed and the cells adhering to the underside of the membranes were fixed, stained, and counted under a microscope based on at least five random fields.

### Western Blot

Protein quantification by Western blot was performed as we previously described (Chang et al., 2017). Briefly, total proteins were extracted and separated by sodium dodecyl sulfate-polyacrylamide gel electrophoresis (SDS-PAGE) and then transferred onto PVDF membrane (Millipore). After incubation with primary antibodies against ITGB1 (#9699, 1;1000, Cell Signaling Technology, Danvers, MA, United States), EZH2 (17-662, 1;1000, Millipore), MYC (#18583, 1;1000, Cell Signaling Technology), β-catenin (#8480, 1;1000, Cell Signaling Technology), histone H3 (#14269, 1;1000, Cell Signaling Technology), or GAPDH (#5174, 1;1000, Cell Signaling Technology), the membranes were further incubated with Goat anti-Rabbit or Goat anti-Mouse secondary antibodies.

### RNA Fluorescence *in situ* Hybridization

For *in situ* detection of ITGB1-DT in LUAD cells, the probes complementary to ITGB1-DT were synthesized by Advanced Cell Diagnostics (ACD, Newark, CA, United States). The hybridization and fluorescence detection were conducted using the RNAscope Fluorescent Multiplex Detection Kit (ACD) following the manufacturer’s manual. Confocal laser scanning microscopy (Leica, Wetzlar, Germany) was used to detect the subcellular localization of ITGB1-DT in LUAD cells.

### Isolation of Cytoplasmic and Nuclear RNA

Fractionation of cytoplasmic and nuclear RNA in A549 cells was conducted using the Cytoplasmic and Nuclear RNA Purification Kit (Norgen, Belmont, CA, United States). Fractionated RNA from the same amounts of cells was used for qRT-PCR analysis.

### RNA Pull-Down

Sense or antisense strand of biotinylated ITGB1-DT was *in vitro* transcribed from pSPT19-ITGB1-DT using the Biotin RNA Labeling Mix (Roche) and T7 or Sp6 RNA polymerase (Roche), respectively. After DNase I treatment for 30 min at 37°C to remove DNA templates, the *in vitro* transcribed RNA was purified using the RNeasy Mini Kit (Qiagen, Hilden, Germany). Three micrograms per reaction of purified RNA was denatured for 5 min at 65°C in RNA Structure buffer and slowly cooled down to room temperature. Then, folded RNA was incubated with 1 mg of whole-cell lysates from A549 cells at 25°C for 1 h. The complexes were further isolated by the streptavidin agarose beads (Invitrogen). To harvest the proteins, 50 ml of 1 × SDS loading buffer was added and boiled for 10 min. Retrieved protein was detected by Western blot.

### RNA Immunoprecipitation (RIP)

RNA immunoprecipitation assays were conducted in A549 cells using the Magna RIP RNA-Binding Protein Immunoprecipitation Kit (Millipore) and a primary antibody against EZH2 (17-662, Millipore) according to the manufacturer’s manual. Retrieved RNA was detected by qRT-PCR.

### Chromatin Immunoprecipitation (ChIP)

Chromatin immunoprecipitation assays were conducted in indicated LUAD cells using the EZ-Magna ChIP Kit (17-10086, Millipore) and primary antibodies against EZH2 (17-662, Millipore), H3K27me3 (17-622, Millipore), or MYC (#18583, Cell Signaling Technology). Retrieved DNA was detected by qRT-PCR with the primers 5′-ATCCTCCGCCTCCTCCTA-3′ (forward) and 5′-TCCTCTGCGCGTCTGATC-3′ (reverse) for *ITGB1* promoter, 5′-CCCGTTTGTCCACTGATGTC-3′ (forward) and 5′-CTTAGCCAGTTCCCTTCCAG-3′ (reverse) for *ITGB1-DT* promoter, and 5′-CTCTAAGTCCTAACCCCTCT-3′ (forward) and 5′-CCACATACGTCCTTTACGATA-3′ (reverse) for negative control.

### Luciferase Reporter Assay

To measure the transcriptional activity of β-catenin, TOPFlash (β-catenin-LEF/TCF sensitive luciferase reporter vector) or FOPFlash (LEF/TCF binding site mutated luciferase reporter vector) was co-transfected with pRL-TK (Promega, Madison, WI, United States) into indicated LUAD cells. pRL-TK expressed Renilla luciferase and was used as endogenous control. The luciferase activities were measured using the Dual-luciferase Reporter Assay System (Promega) 48 h after transfection.

### *In vivo* Xenograft Studies

For *in vivo* lung metastasis assays, 2 × 10^6^ indicated LUAD cells labeled by luciferase were injected into the lateral tail veins of female BABL/c athymic nude mice (age 4–5 weeks). Seven weeks later, the activities of luciferase in lungs were detected and captured under IVIS Lumina II (Xenogen, Hopkinton, MA, United States). The mice were sacrificed and the lungs were excised, fixed in formalin, and subjected to hematoxylin and eosin (H&E) staining to detect lung metastatic nodules. The animal experimental procedures were approved by the Animal Ethics Committee of Xiangya Hospital.

### Statistical Analysis

All statistical analyses were conducted using GraphPad Prism v6.0 (GraphPad Software Inc., La Jolla, CA, United States). For comparison of the differences between two groups, Wilcoxon signed rank test (non-parametric) or Student’s *t*-test (parametric) was performed. For comparison of the differences between more than two groups, one-way ANOVA followed by Dunnett’s multiple comparisons test, or Kruskal–Wallis test (non-parametric one-way ANOVA) followed by Dunn’s multiple comparisons test was performed. Kaplan–Meier survival analyses were performed using Log-rank test. Gene expression correlation analyses in clinical specimens were performed using Spearman correlation analysis. Analyses of the correlation between gene expression and clinicopathological features were performed using chi-square test. *P*-values < 0.05 were considered statistically significant.

## Results

### ITGB1-DT Was a Poor Prognosis-Related lncRNA in LUAD

**FIGURE 1:**
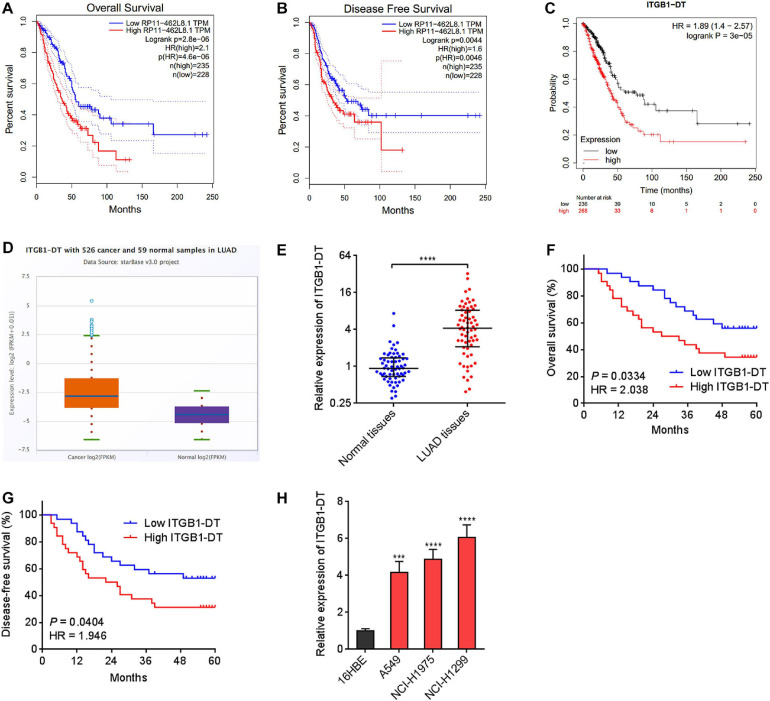
ITGB1-DT was upregulated in LUAD and correlated with poor prognosis of LUAD patients. **(A)** The correlation between ITGB1-DT (RP11-462L8.1) expression and overall survival from TCGA LUAD dataset analyzed by GEPIA. **(B)** The correlation between ITGB1-DT (RP11-462L8.1) expression and disease-free survival from TCGA LUAD dataset analyzed by GEPIA. **(C)** The correlation between ITGB1-DT expression and overall survival in LUAD based on Kaplan–Meier Plotter dataset. **(D)** ITGB1-DT expression in LUAD tissues and normal tissues from TCGA dataset analyzed by starBase. **(E)** ITGB1-DT expression in 64 pairs of LUAD tissues and matched normal lung tissues was measured by qRT-PCR. ****P < 0.0001 by Wilcoxon signed rank test. **(F)** Kaplan–Meier survival analyses of the correlation between ITGB1-DT expression and overall survival in 64 LUAD patients. *P* = 0.0334, HR = 2.038 by Log-rank test. **(G)** Kaplan–Meier survival analyses of the correlation between ITGB1-DT expression and disease-free survival in 64 LUAD patients. *P* = 0.0404, HR = 1.946 by Log-rank test. **(H)** ITGB1-DT expression in normal bronchial epithelial cell line 16HBE and LUAD cell lines A549, NCI-H1975, and NCI-H1299 was measured by qRT-PCR. Results are presented as mean ± SD of three independent experiments. ***P < 0.001, ****P < 0.0001 by one-way ANOVA followed by Dunnett’s multiple comparisons test.

To identify the lncRNAs associated with overall survival in LUAD, we analyzed TCGA LUAD dataset using the online tool Gene Expression Profiling Interactive Analysis (GEPIA)^1^. The genes associated with overall survival of LUAD were shown in Supplementary Table 1. The most significant lncRNA was RP11-462L8.1, which is also named as ITGB1-DT. Through analyzing TCGA LUAD dataset using GEPIA, we found that high expression of RP11-462L8.1 (ITGB1-DT) was not only correlated with poor overall survival, but also correlated with poor disease-free survival in LUAD (Figures 1A,B). Another online database, Kaplan–Meier Plotter^2^, also revealed that high expression of ITGB1-DT was correlated with poor survival in LUAD (Figure 1C). Analysis of ITGB1-DT expression in 526 LUAD tissues and 59 normal tissues from TCGA dataset by starBase^3^ revealed that the expression of ITGB1-DT was increased in LUAD tissues compared with normal tissues (Figure 1D). In addition, we measured ITGB1-DT expression in 64 pairs of LUAD tissues and matched normal lung tissues. Consistent with TCGA dataset, our results showed that ITGB1-DT was upregulated in LUAD tissues compared with paired normal tissues (Figure 1E). Kaplan–Meier survival analyses also revealed that high ITGB1-DT expression was correlated with poor overall survival and disease-free survival in these 64 LUAD patients (Figures 1F,G). Analyzing the correlations between ITGB1-DT expression and clinicopathological features of LUAD revealed that high ITGB1-DT expression was correlated with local invasion, lymphatic metastasis, and advanced TNM stages (Table 1). To further elucidate the expression pattern of ITGB1-DT in LUAD cells, we measured ITGB1-DT expression in normal bronchial epithelial cell line 16HBE and LUAD cell lines A549, NCI-H1975, and NCI-H1299. The results revealed that ITGB1-DT was also unregulated in LUAD cells compared with normal bronchial epithelial cell line (Figure 1H). The coding potential of ITGB1-DT was calculated using three *in silico* tools, Coding Potential Assessment Tool (CPAT)^4^, Coding Potential Calculator (CPC)^5^, and PhyloCSF. The CPAT and CPC scores of ITGB1-DT were 0.00838 and -1.00977, respectively, both of which indicated ITGB1-DT as non-coding. Furthermore, the PhyloCSF from UCSC Genome Browser^6^ also identified the non-coding nature of ITGB1-DT. Therefore, these findings characterized ITGB1-DT as an upregulated and poor prognosis-related lncRNA in LUAD.

### ITGB1-DT Facilitated LUAD Cellular Proliferation, Migration, and Invasion

**FIGURE 2:**
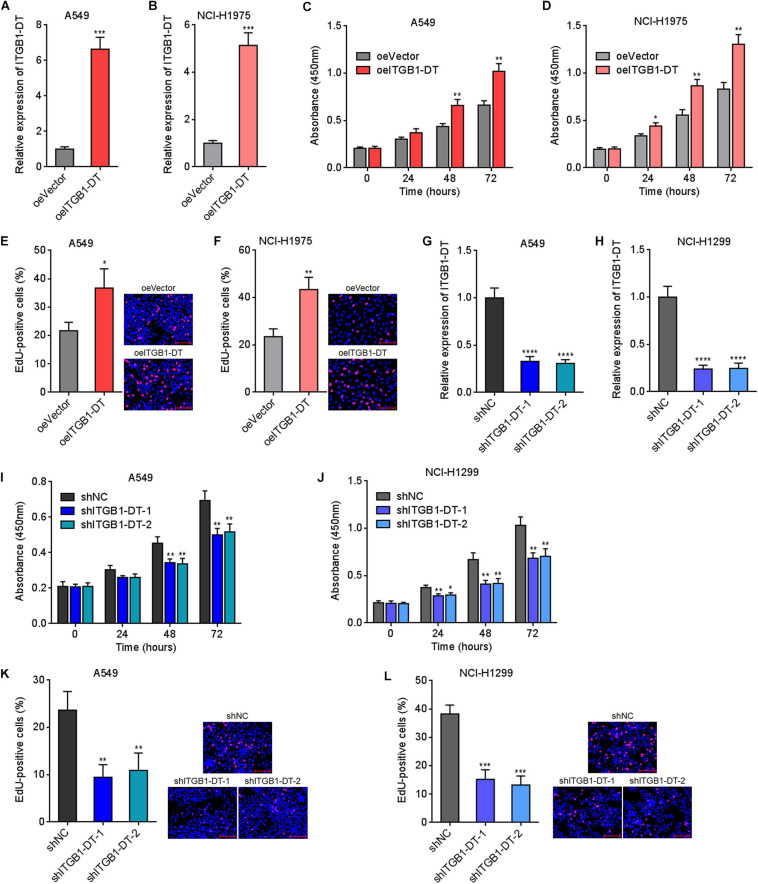
ITGB1-DT facilitated LUAD cellular proliferation. **(A)** ITGB1-DT expression in A549 cells with ITGB1-DT stable overexpression was measured by qRT-PCR. **(B)** ITGB1-DT expression in NCI-H1975 cells with ITGB1-DT stable overexpression was measured by qRT-PCR. **(C)** Cell proliferation of A549 cells with ITGB1-DT stable overexpression was measured by CCK-8 assays. **(D)** Cell proliferation of NCI-H1975 cells with ITGB1-DT stable overexpression was measured by CCK-8 assays. **(E)** Cell proliferation of A549 cells with ITGB1-DT stable overexpression was measured by EdU incorporation assays. Red color indicates EdU-positive cells. Scale bar, 100 μm. **(F)** Cell proliferation of NCI-H1975 cells with ITGB1-DT stable overexpression was measured by EdU incorporation assays. Red color indicates EdU-positive cells. Scale bar, 100 μm. **(G)** ITGB1-DT expression in A549 cells with ITGB1-DT stable silencing was measured by qRT-PCR. **(H)** ITGB1-DT expression in NCI-H1299 cells with ITGB1-DT stable silencing was measured by qRT-PCR. **(I)** Cell proliferation of A549 cells with ITGB1-DT stable silencing was measured by CCK-8 assays. **(J)** Cell proliferation of NCI-H1299 cells with ITGB1-DT stable silencing was measured by CCK-8 assays. **(K)** Cell proliferation of A549 cells with ITGB1-DT stable silencing was measured by EdU incorporation assays. Red color indicates EdU-positive cells. Scale bar, 100 μm. **(L)** Cell proliferation of NCI-H1299 cells with ITGB1-DT stable silencing was measured by EdU incorporation assays. Red color indicates EdU-positive cells. Scale bar, 100 μm. Results are presented as mean ± SD of three independent experiments. *P < 0.05, **P < 0.01, ***P < 0.001, ****P < 0.0001 by Student’s *t*-test **(A–F)**, or one-way ANOVA followed by Dunnett’s multiple comparisons test **(G–L)**.

Due to the significant correlation between ITGB1-DT and poor prognosis in LUAD, we next investigated the potential roles of ITGB1-DT in LUAD. ITGB1-DT stably overexpressed LUAD cells were constructed via stable transfection of ITGB1-DT overexpression vector into A549 and NCI-H1975 cells (Figures 2A,B). CCK-8 assays showed that cellular proliferation rates of A549 and NCI-H1975 cells with ITGB1-DT overexpression were remarkably quicker than that of control A549 and NCI-H1975 cells (Figures 2C,D). EdU incorporation assays showed that A549 and NCI-H1975 cells with ITGB1-DT overexpression had more EdU-positive cells than control A549 and NCI-H1975 cells (Figures 2E,F), also suggesting that ITGB1-DT facilitated LUAD cellular proliferation. Moreover, ITGB1-DT stably silenced LUAD cells were constructed via stable infection of two independent ITGB1-DT specific shRNA lentiviruses into A549 and NCI-H1299 cells (Figures 2G,H). CCK-8 assays showed that cellular proliferation rates of A549 and NCI-H1299 cells with ITGB1-DT silencing were remarkably slower than that of control A549 and NCI-H1299 cells (Figures 2I,J). EdU incorporation assays revealed that A549 and NCI-H1299 cells with ITGB1-DT silencing had less EdU-positive cells than control A549 and NCI-H1299 cells (Figures 2K,L), suggesting that ITGB1-DT silencing repressed LUAD cellular proliferation. Thus, these findings demonstrated that ITGB1-DT facilitated LUAD cellular proliferation.

**FIGURE 3:**
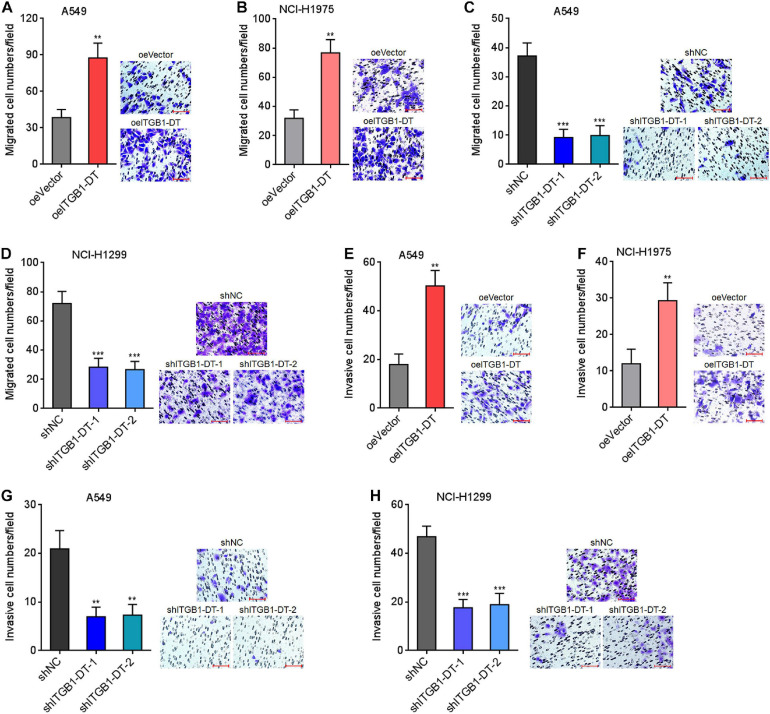
ITGB1-DT facilitated LUAD cellular migration and invasion. **(A)** Cell migration of A549 cells with ITGB1-DT stable overexpression was measured by transwell migration assays. **(B)** Cell migration of NCI-H1975 cells with ITGB1-DT stable overexpression was measured by transwell migration assays. **(C)** Cell migration of A549 cells with ITGB1-DT stable silencing was measured by transwell migration assays. **(D)** Cell migration of NCI-H1299 cells with ITGB1-DT stable silencing was measured by transwell migration assays. **(E)** Cell invasion of A549 cells with ITGB1-DT stable overexpression was measured by transwell invasion assays. **(F)** Cell invasion of NCI-H1975 cells with ITGB1-DT stable overexpression was measured by transwell invasion assays. **(G)** Cell invasion of A549 cells with ITGB1-DT stable silencing was measured by transwell invasion assays. **(H)** Cell invasion of NCI-H1299 cells with ITGB1-DT stable silencing was measured by transwell invasion assays. Results are presented as mean ± SD of three independent experiments. **P < 0.01, ***P < 0.001 by Student’s *t*-test **(A,B,E,F)**, or one-way ANOVA followed by Dunnett’s multiple comparisons test **(C,D,G,H)**. Scale bar, 100 μm.

Next, the potential roles of ITGB1-DT in LUAD cellular migration and invasion were investigated using transwell migration and invasion assays, respectively. Transwell migration assays showed that A549 and NCI-H1975 cells with ITGB1-DT overexpression had more migrated cells than that of control A549 and NCI-H1975 cells (Figures 3A,B). Conversely, A549 and NCI-H1299 cells with ITGB1-DT silencing had less migrated cells than that of control A549 and NCI-H1299 cells (Figures 3C,D). Transwell invasion assays showed that A549 and NCI-H1975 cells with ITGB1-DT overexpression had more invasive cells than control A549 and NCI-H1975 cells (Figures 3E,F). Conversely, A549 and NCI-H1299 cells with ITGB1-DT silencing had less invasive cells than control A549 and NCI-H1299 cells (Figures 3G,H). Epithelial–mesenchymal transition (EMT) has been frequently reported to be involved in cellular migration and invasion (Yuan et al., 2014). Thus, the potential effects of ITGB1-DT on LUAD EMT were investigated. A549 cells with ITGB1-DT overexpression showed reduced expression of epithelial marker E-cadherin and increased expression of mesenchymal markers N-cadherin and Vimentin compared with control A549 cells (Supplementary Figure 1A), indicating that ITGB1-DT overexpression induced EMT. Conversely, A549 cells with ITGB1-DT silencing showed increased expression of E-cadherin and reduced expression of N-cadherin and Vimentin compared with control A549 cells (Supplementary Figure 1B), indicating that ITGB1-DT silencing repressed EMT. Collectively, these findings demonstrated that ITGB1-DT facilitated LUAD cellular migration, invasion, and EMT.

### ITGB1-DT Epigenetically Upregulated ITGB1 Expression

**FIGURE 4:**
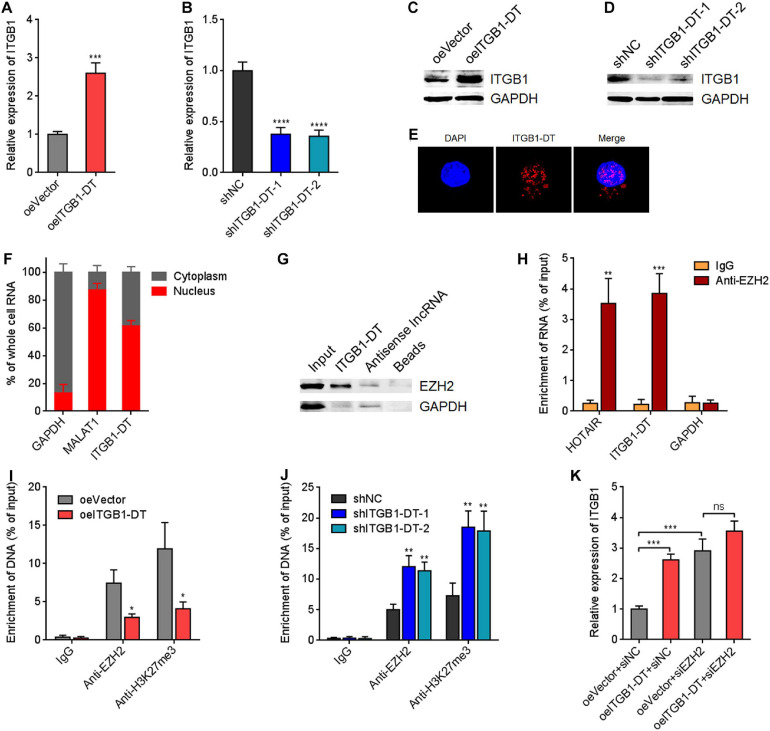
ITGB1-DT upregulated ITGB1 expression via binding EZH2. **(A)** ITGB1 mRNA levels in A549 cells with ITGB1-DT stable overexpression was measured by qRT-PCR. **(B)** ITGB1 mRNA levels in NCI-H1299 cells with ITGB1-DT stable silencing was measured by qRT-PCR. **(C)** ITGB1 protein levels in A549 cells with ITGB1-DT stable overexpression was measured by Western blot. **(D)** ITGB1 protein levels in NCI-H1299 cells with ITGB1-DT stable silencing was measured by Western blot. **(E)** RNA FISH indicated the mainly nuclear localization of ITGB1-DT in A549 cells. **(F)** Biochemical fractionation of A549 cells, followed by qRT-PCR to detect the subcellular localization of ITGB1-DT. GAPDH and MALAT1 were used as cytoplasmic and nuclear controls, respectively. **(G)** RNA pull-down assays with sense or antisense strand of biotinylated ITGB1-DT, followed by Western blot to detect the proteins binding of EZH2 to ITGB1-DT. GAPDH was used as negative control. **(H)** RIP assays with EZH2-specific antibody were conducted in A549 cells to detect the binding of ITGB1-DT to EZH2. HOTAIR and GAPDH were used as positive and negative controls, respectively. **(I)** ChIP assays with EZH2- and H3K27me3-specific antibodies were conducted in A549 cells with ITGB1-DT overexpression or control. The enrichment of *ITGB1* promoter region was detected by qRT-PCR. **(J)** ChIP assays with EZH2- and H3K27me3-specific antibodies were conducted in NCI-H1299 cells with ITGB1-DT silencing or control. The enrichment of *ITGB1* promoter region was detected by qRT-PCR. **(K)** After transient transfection of EZH2 siRNAs into A549 cells with ITGB1-DT stable overexpression or control, ITGB1 mRNA levels were measured by qRT-PCR. Results are presented as mean ± SD of three independent experiments. *P < 0.05, **P < 0.01, ***P < 0.001, ****P < 0.0001; ns, not significant, by Student’s *t*-test **(A,H,I)**, or one-way ANOVA followed by Dunnett’s multiple comparisons test **(B,J)**. In panel **K**, the comparisons of oeITGB1 + siNC and oeVector + siEZH2 with oeVector + siNC were performed using one-way ANOVA followed by Dunnett’s multiple comparisons test; the comparisons of oeITGB1 + siEZH2 with oeVector + siEZH2 were performed using Student’s *t*-test.

To gain insight into the mechanisms underlying the oncogenic roles of ITGB1-DT in LUAD, we focused on the potential regulatory effects of ITGB1-DT on ITGB1 for two reasons: (1) *ITGB1-DT* is located at the antisense strand of *ITGB1*; (2) ITGB1 has been reported to exert oncogenic roles in multiple cancers, including LUAD (Wang et al., 2019; Zhang C. et al., 2020). Intriguingly, our results revealed that A549 cells with ITGB1-DT overexpression had higher ITGB1 mRNA expression levels than control A549 cells (Figure 4A). Conversely, NCI-H1299 cells with ITGB1-DT silencing had lower ITGB1 mRNA expression levels than control NCI-H1299 cells (Figure 4B). Consistent with the changes of ITGB1 mRNA levels, ITGB1 protein expression levels were also significantly increased in A549 cells with ITGB1-DT overexpression compared with control A549 cells (Figure 4C). ITGB1 protein expression levels were significantly reduced in NCI-H1299 cells with ITGB1-DT silencing compared with control NCI-H1299 cells (Figure 4D). To further elucidate the potential regulatory mechanisms of ITGB1-DT on ITGB1, we detected the subcellular localization of ITGB1-DT using RNA FISH. The results revealed that ITGB1-DT was localized in both the nucleus and cytoplasm, but more enrichment in nucleus (Figure 4E). Biochemical fractionation of A549 cells, followed by qRT-PCR, also demonstrated that ITGB1-DT was mainly localized in nucleus (Figure 4F). Multiple nuclear lncRNAs were reported to regulate chromatin architecture via binding epigenetic regulators (Bai et al., 2020; Zhu et al., 2016). To investigate whether ITGB1-DT modulated ITGB1 in a similar manner, we predicted the potential interaction between ITGB1-DT and epigenetic regulators using the online tool RNA-Protein Interaction Prediction (RPISeq)^7^. Intriguingly, histone lysine N-methyltransferase EZH2, a major component of polycomb repressive complex 2 (PRC2), was identified to interact with ITGB1-DT with an interaction probability score of 0.86 (> 0.5 was considered positive). To elucidate whether ITGB1-DT binds to EZH2 in LUAD cells, we conducted RNA pull-down assays using sense or antisense strand of biotinylated ITGB1-DT. The enriched proteins were detected by Western blot. As presented in Figure 4G, EZH2 was specifically enriched in the ITGB1-DT group, supporting the potential interaction between EZH2 and ITGB1-DT. Furthermore, RIP assays using EZH2 specific antibody showed that ITGB1-DT was specifically enriched in the EZH2 antibody group (Figure 4H), supporting the interaction between ITGB1-DT and EZH2. As a histone methyltransferase, EZH2 induced trimethylation at lysine 27 of histone H3 (H3K27me3), leading to the repression of target genes (Hanniford et al., 2020). Thus, we further investigated the potential effects of ITGB1-DT on the modulation of ITGB1 by EZH2. ChIP assays revealed that the binding of EZH2 to *ITGB1* promoter and H3K27me3 levels at *ITGB1* promoter were both reduced in A549 cells with ITGB1-DT overexpression compared with control A549 cells (Figure 4I). Conversely, the binding of EZH2 to *ITGB1* promoter and H3K27me3 levels at *ITGB1* promoter were both increased in NCI-H1299 cells with ITGB1-DT silencing compared with control NCI-H1299 cells (Figure 4J). To determine whether EZH2 is the critical mediator of the roles of ITGB1-DT in upregulating ITGB1, EZH2 was silenced in A549 cells with ITGB1-DT overexpression or control. EZH2 depletion upregulated ITGB1 expression (Figure 4K). At the context of EZH2 depletion, ITGB1-DT overexpression did not upregulate ITGB1 expression (Figure 4K), indicating that EZH2 at least partially contributed to the upregulation of ITGB1 by ITGB1-DT. Collectively, these findings suggested that ITGB1-DT interacted with EZH2 and reduced the binding of EZH2 to *ITGB1* promoter, leading to the downregulation of repressive chromatin marker H3K27me3 at *ITGB1* promoter and the upregulation of ITGB1 expression.

### The Expression of ITGB1 Was Positively Associated With That of ITGB1-DT in LUAD Tissues and Also Indicated Poor Outcome of LUAD Patients

**FIGURE 5:**
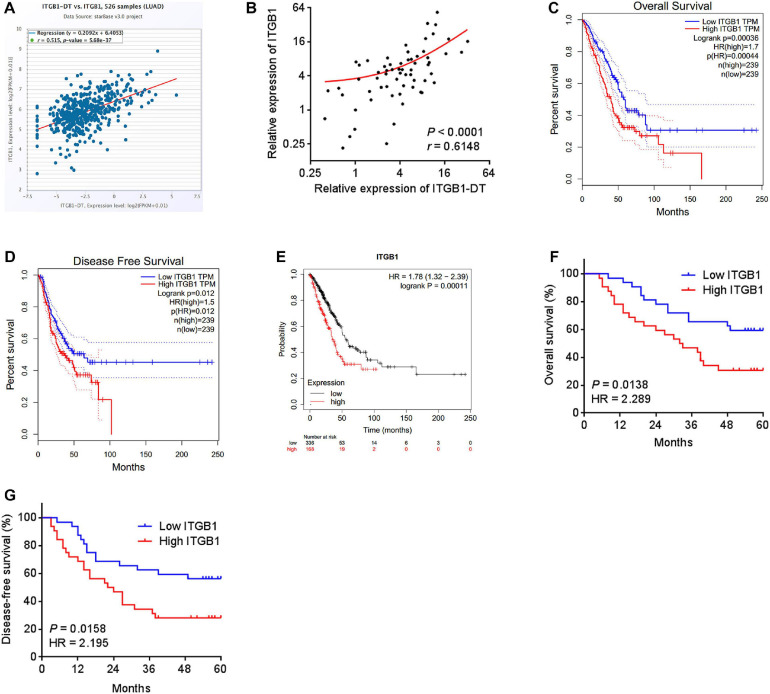
The expression of ITGB1 was positively associated with that of ITGB1-DT, and also related to poor prognosis in LUAD. **(A)** The correlation between ITGB1 and ITGB1-DT expression levels from TCGA LUAD dataset analyzed by starBase. **(B)** The correlation between ITGB1 and ITGB1-DT expression levels in 64 LUAD tissues. *P* < 0.0001, *r* = 0.6148 by Spearman correlation analysis. **(C)** The correlation between ITGB1 expression and overall survival from TCGA LUAD dataset analyzed by GEPIA. **(D)** The correlation between ITGB1 expression and disease-free survival from TCGA LUAD dataset analyzed by GEPIA. **(E)** The correlation between ITGB1 expression and overall survival in LUAD based on Kaplan–Meier Plotter dataset. **(F)** Kaplan–Meier survival analyses of the correlation between ITGB1 expression and overall survival in 64 LUAD patients. *P* = 0.0138, HR = 2.289 by Log-rank test. **(G)** Kaplan–Meier survival analyses of the correlation between ITGB1 expression and disease-free survival in 64 LUAD patients. *P* = 0.0158, HR = 2.195 by Log-rank test.

To elucidate whether the upregulation of ITGB1 by ITGB1-DT also exists *in vivo*, the correlation between ITGB1 expression and ITGB1-DT expression in TCGA LUAD tissues was investigated using the online tool starBase (see text footnote 3). The results revealed that the expression of ITGB1-DT was remarkably positively correlated with the expression of ITGB1 in TCGA LUAD tissues (Figure 5A). Furthermore, in our own cohort, we also found the remarkable positive correlation between ITGB1 and ITGB1-DT expression in LUAD tissues (Figure 5B). Consistent with ITGB1-DT, high expression of ITGB1 was also correlated with poor overall survival and disease-free survival in TCGA LUAD dataset analyzed by GEPIA (see text footnote 1) (Figures 5C,D). The correlation between high ITGB1 expression and poor survival in LUAD was also found in another online database, Kaplan–Meier Plotter (see text footnote 2) (Figure 5E). In our own cohort, we also found that high ITGB1 expression was correlated with poor overall survival and disease-free survival in LUAD (Figures 5F,G). Therefore, these findings demonstrated that the expression of ITGB1 was positively correlated with that of ITGB1-DT in LUAD tissues, and high ITGB1 expression was also correlated with poor outcome of LUAD patients, supporting the positive regulation of ITGB1 by ITGB1-DT *in vivo*.

### ITGB1-DT Activated Wnt/β-Catenin/MYC Axis

**FIGURE 6:**
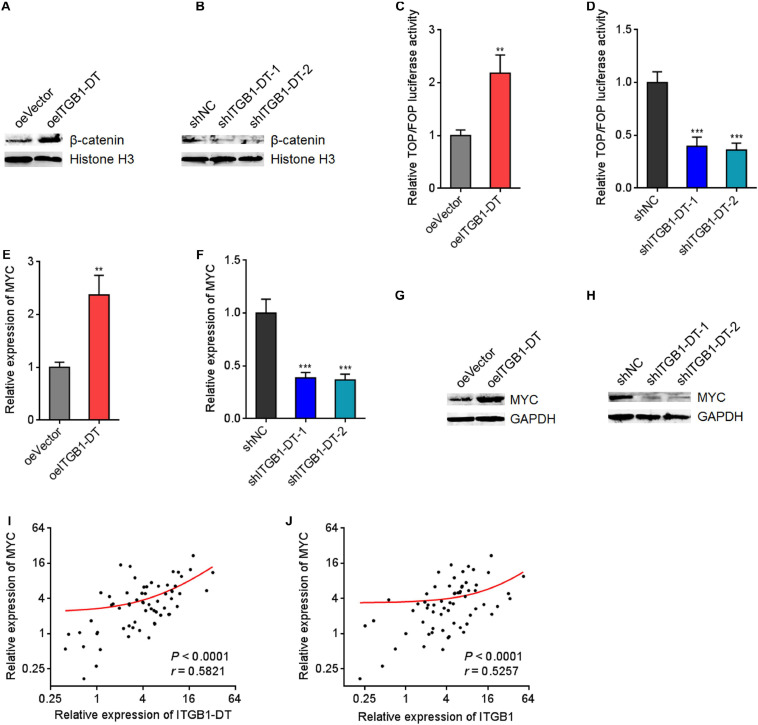
ITGB1-DT activated Wnt/β-catenin/MYC axis. **(A)** Nuclear β-catenin levels in A549 cells with ITGB1-DT stable overexpression was measured by Western blot. **(B)** Nuclear β-catenin levels in NCI-H1299 cells with ITGB1-DT stable silencing was measured by Western blot. **(C)** TOPFlash or FOPFlash vectors were transfected into A549 cells with ITGB1-DT stable overexpression or control. Forty-eight hours later, the TOPFlash/FOPFlash luciferase activates were measured. **(D)** TOPFlash or FOPFlash vectors were transfected into NCI-H1299 cells with ITGB1-DT stable silencing or control. Forty-eight hours later, the TOPFlash/FOPFlash luciferase activates were measured. **(E)** MYC mRNA levels in A549 cells with ITGB1-DT stable overexpression was measured by qRT-PCR. **(F)** MYC mRNA levels in NCI-H1299 cells with ITGB1-DT stable silencing was measured by qRT-PCR. **(G)** MYC protein levels in A549 cells with ITGB1-DT stable overexpression was measured by Western blot. **(H)** MYC protein levels in NCI-H1299 cells with ITGB1-DT stable silencing was measured by Western blot. **(I)** The correlation between MYC and ITGB1-DT expression levels in 64 LUAD tissues. *P* < 0.0001, *r* = 0.5821 by Spearman correlation analysis. **(J)** The correlation between MYC and ITGB1 expression levels in 64 LUAD tissues. *P* < 0.0001, *r* = 0.5257 by Spearman correlation analysis. For C–F, Results are presented as mean ± SD of three independent experiments. **P < 0.01, ***P < 0.001 by Student’s *t*-test **(C,E)**, or one-way ANOVA followed by Dunnett’s multiple comparisons test **(D,F)**.

Because ITGB1 was reported to activate Wnt/β-catenin pathway in multiple cancers, including LUAD, we next investigated the potential influences of ITGB1-DT on Wnt/β-catenin pathway. Nuclear β-catenin levels were upregulated in A549 cells with ITGB1-DT overexpression compared with control A549 cells (Figure 6A), and downregulated in NCI-H1299 cells with ITGB1-DT silencing compared with control NCI-H1299 cells (Figure 6B). Additionally, the luciferase activities of TOPFlash/FOPFlash were detected to evaluate the activation of Wnt/β-catenin pathway. TOPFlash/FOPFlash ratio was increased in A549 cells with ITGB1-DT overexpression compared with control A549 cells (Figure 6C) and downregulated in NCI-H1299 cells with ITGB1-DT silencing compared with control NCI-H1299 cells (Figure 6D). These results demonstrated that ITGB1-DT activated Wnt/β-catenin pathway in LUAD. MYC is a well-known Wnt/β-catenin downstream target and exhibits oncogenic roles in various cancers (Zhen-Hua et al., 2020). Thus, we further detected the effects of ITGB1-DT on MYC expression. Our results revealed that MYC mRNA levels were increased in A549 cells with ITGB1-DT overexpression compared with control A549 cells (Figure 6E) and downregulated in NCI-H1299 cells with ITGB1-DT silencing compared with control NCI-H1299 cells (Figure 6F). Consistently, MYC protein levels were increased in A549 cells with ITGB1-DT overexpression compared with control A549 cells (Figure 6G) and downregulated in NCI-H1299 cells with ITGB1-DT silencing compared with control NCI-H1299 cells (Figure 6H). In addition, ITGB1-DT and ITGB1 expression levels were both remarkably positively correlated with MYC expression levels in LUAD tissues (Figures 6I,J), supporting the regulation of MYC by ITGB1-DT and ITGB1. Collectively, these findings demonstrated that ITGB1-DT activated Wnt/β-catenin/MYC axis.

### MYC Directly Activated ITGB1-DT Transcription, and ITGB1-DT/ITGB1/Wnt/β-Catenin/MYC Formed a Positive Feedback Loop

**FIGURE 7:**
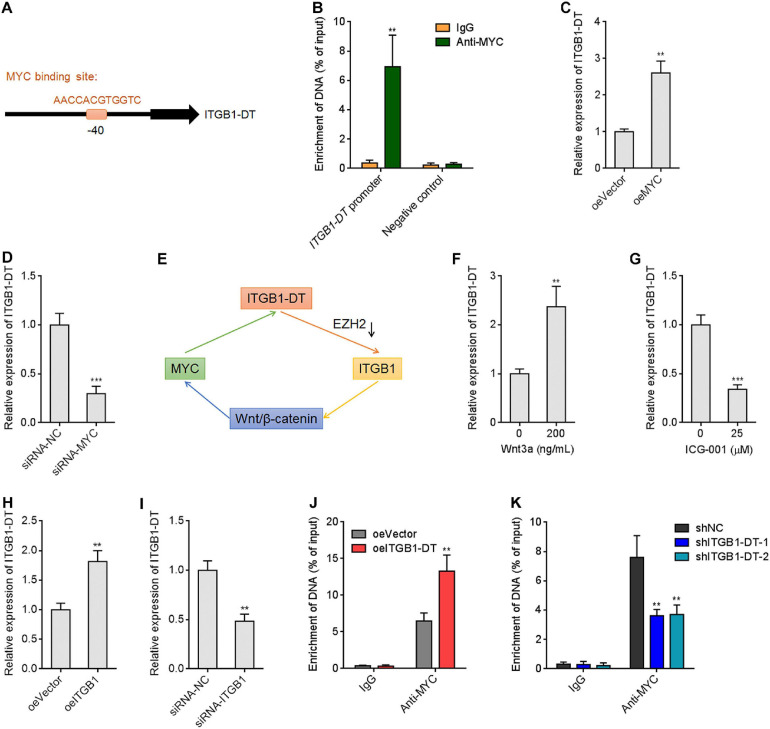
MYC directly activated *ITGB1-DT* transcription, and ITGB1-DT/ITGB1/Wnt/β-catenin/MYC formed a positive feedback loop. **(A)** The predicted MYC binding site in the *ITGB1-DT* promoter. **(B)** ChIP assays with MYC specific antibody were conducted in A549 cells. The enrichment of *ITGB1-DT* promoter region was detected by qRT-PCR. **(C)** ITGB1-DT expression in A549 cells with MYC overexpression was measured by qRT-PCR. **(D)** ITGB1-DT expression in A549 cells with MYC silencing was measured by qRT-PCR. **(E)** Schematic diagram of the ITGB1-DT/ITGB1/Wnt/β-catenin/MYC positive feedback loop. **(F)** ITGB1-DT expression in A549 cells after 200 ng/ml Wnt3a treatment for 24 h. **(G)** ITGB1-DT expression in A549 cells after 25 μM ICG-001 treatment for 24 h. **(H)** ITGB1-DT expression in A549 cells with ITGB1 overexpression was measured by qRT-PCR. **(I)** ITGB1-DT expression in A549 cells with ITGB1 silencing was measured by qRT-PCR. **(J)** ChIP assays with MYC specific antibodies were conducted in A549 cells with ITGB1-DT overexpression or control. The enrichment of *ITGB1-DT* promoter region was detected by qRT-PCR. **(K)** ChIP assays with MYC specific antibodies were conducted in NCI-H1299 cells with ITGB1-DT silencing or control. The enrichment of *ITGB1-DT* promoter region was detected by qRT-PCR. Results are presented as mean ± SD of three independent experiments. **P < 0.01, ***P < 0.001 by Student’s *t*-test **(B,C,D,F,G,H,I,J)**, or one-way ANOVA followed by Dunnett’s multiple comparisons test **(K)**.

Due to the significant positive correlation between ITGB1-DT and MYC in LUAD, we further investigated whether MYC also regulates ITGB1-DT. Intriguingly, a MYC binding site was identified in the promoter region of *ITGB1-DT* by *in silico* tool JASPAR^8^ with a score of 15.5622 (Figure 7A). To investigate whether MYC binds to *ITGB1-DT* promoter region, ChIP assays were performed using MYC specific antibody. The ChIP results revealed that MYC directly bound *ITGB1-DT* promoter, but not a distal negative control region (Figure 7B). ITGB1-DT expression was increased after MYC overexpression and reduced after MYC silencing (Figures 7C,D), which suggested that MYC activated ITGB1-DT expression. Thus, ITGB1-DT/ITGB1/Wnt/β-catenin/MYC may form a positive feedback loop in LUAD (Figure 7E). Activation of Wnt/β-catenin pathway by Wnt3a upregulated ITGB1-DT expression (Figure 7F). In contrast, repression of Wnt/β-catenin pathway by ICG-001 downregulated ITGB1-DT expression (Figure 7G). As expected, ITGB1 overexpression upregulated ITGB1-DT expression (Figure 7H), and ITGB1 silencing downregulated ITGB1-DT expression (Figure 7I). Overexpression of ITGB1-DT increased the binding of MYC to *ITGB1-DT* promoter (Figure 7J), and ITGB1-DT silencing reduced the binding of MYC to *ITGB1-DT* promoter (Figure 7K). These findings supported the ITGB1-DT/ITGB1/Wnt/β-catenin/MYC positive feedback loop.

### The Oncogenic Roles of ITGB1-DT in LUAD *in vitro* and *in vivo* Were Dependent on the Positive Modulation of ITGB1/Wnt/β-Catenin

**FIGURE 8:**
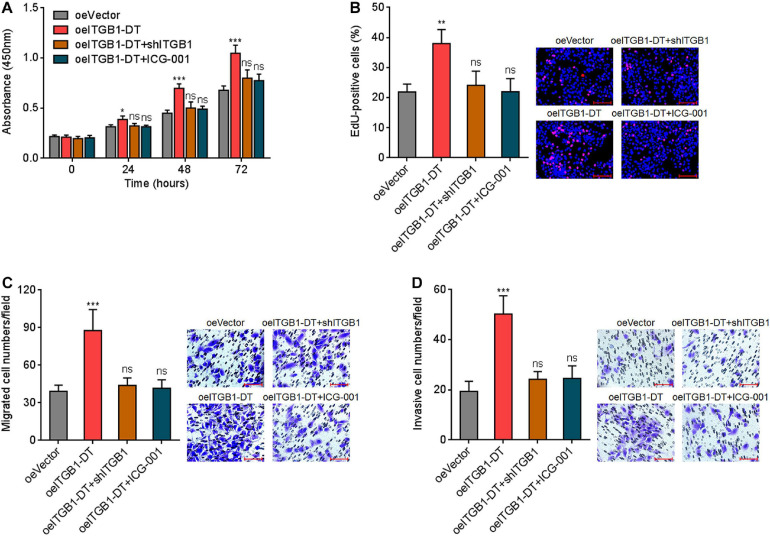
Repression of ITGB1 or Wnt/β-catenin pathway reversed the roles of ITGB1-DT in promoting LUAD cellular proliferation, migration, and invasion. **(A)** ITGB1 was depleted in A549 cells with ITGB1-DT overexpression. A549 cells with ITGB1-DT overexpression were treated with 25 μM ICG-001. Cell proliferation of these indicated A549 cells was detected by CCK-8 assays. **(B)** Cell proliferation of these indicated A549 cells was detected by EdU incorporation assays. Red color indicates EdU-positive cells. Scale bar, 100 μm. **(C)** Cell migration of these indicated A549 cells was detected by transwell migration assays. Scale bar, 100 μm. **(D)** Cell invasion of these indicated A549 cells was detected by transwell invasion assays. Scale bar, 100 μm. Results are presented as mean ± SD of three independent experiments. *P < 0.05, **P < 0.01, ***P < 0.001; ns, not significant, by one-way ANOVA followed by Dunnett’s multiple comparisons test.

To elucidate whether the roles of ITGB1-DT in LUAD were dependent on the positive ITGB1-DT/ITGB1/Wnt/β-catenin/MYC feedback loop, the expression of ITGB1 was silenced in A549 cells with ITGB1-DT overexpression. CCK-8 assays showed that the pro-proliferative roles of ITGB1-DT were reversed by ITGB1 silencing (Figure 8A). Furthermore, repression of Wnt/β-catenin pathway by ICG-001 also reversed the pro-proliferative roles of ITGB1-DT (Figure 8A). EdU incorporation assays showed that the increased EdU-positive cell numbers caused by ITGB1-DT were reversed by ITGB1 silencing or ICG-001 (Figure 8B), which also suggested that ITGB1 silencing or Wnt/β-catenin pathway repression both reversed the pro-proliferative roles of ITGB1-DT in LUAD. Transwell migration assays showed that ITGB1 silencing or ICG-001 both abolished the pro-migratory roles of ITGB1-DT (Figure 8C). Transwell invasion assays showed that ITGB1 silencing or ICG-001 both abolished the pro-invasive roles of ITGB1-DT (Figure 8D).

**FIGURE 9:**
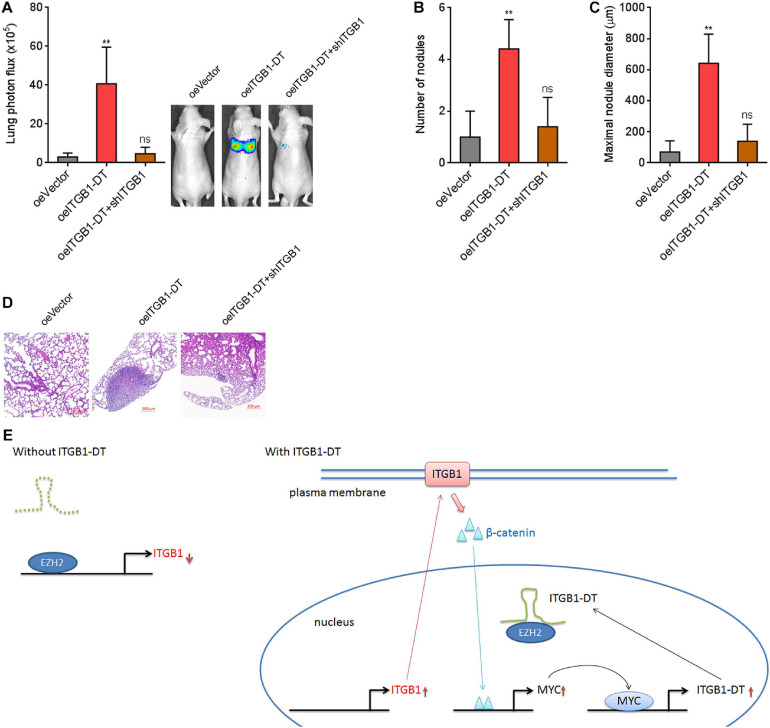
Repression of ITGB1 reversed the roles of ITGB1-DT in promoting LUAD lung metastasis. **(A)** Luciferase-labeled A549 cells with concurrent ITGB1-DT overexpression and ITGB1 depletion were injected into tail vein of nude mice. Lung metastases were detected using bioluminescence analyzing 7 weeks later. **(B)** The numbers of lung metastatic nodules formed by indicated A549 cells were detected by histological examination. **(C)** The maximal metastatic nodular diameters formed by indicated A549 cells were detected by histological examination. **(D)** Representative H&E images of lung metastatic nodules formed by indicated A549 cells. Results are presented as mean ± SD of *n* = 5 mice in each group. **P < 0.01; ns, not significant, by Kruskal–Wallis test (non-parametric one-way ANOVA) followed by Dunn’s multiple comparisons test. **(E)** Schematic diagram of the ITGB1-DT/ITGB1/Wnt/β-catenin/MYC positive feedback loop.

To investigate the potential roles of ITGB1-DT/ITGB1/Wnt/β-catenin/MYC feedback loop *in vivo*, luciferase-labeled A549 cells with concurrent ITGB1-DT overexpression and ITGB1 silencing were injected into tail vein of nude mice. Seven weeks later, lung metastases were detected using bioluminescence analyzing. As shown in Figure 9A, A549 cells with ITGB1-DT overexpression formed more lung metastases compared with control A549 cells. The increased lung metastases caused by ITGB1-DT overexpression was reversed by ITGB1 silencing (Figure 9A). Histological examination of the lungs revealed that the number of lung metastatic nodules formed by A549 cells with ITGB1-DT overexpression was larger than that formed by control A549 cells, which was reversed by ITGB1 silencing (Figure 9B). Consistently, the maximal metastatic nodular diameter formed by A549 cells with ITGB1-DT overexpression was larger than that formed by control A549 cells, which was also reversed by ITGB1 silencing (Figures 9C,D). These findings further demonstrated that ITGB1-DT also exhibited oncogenic roles *in vivo*. The oncogenic roles of ITGB1-DT in LUAD were dependent on the ITGB1-DT/ITGB1/Wnt/β-catenin/MYC positive feedback loop.

## Discussion

Through analyzing the available RNA-seq data of LUAD tissues from TCGA database, we found that ITGB1-DT was the most significantly correlated lncRNAs with overall survival of LUAD. The significant correlation between increased ITGB1-DT expression and poor prognosis of LUAD patients was also found in another available online database, Kaplan–Meier Plotter. Third, the correlation between increased ITGB1-DT expression and poor outcome of LUAD was also verified in our own cohort. Furthermore, ITGB1-DT was found to be highly expressed in LUAD tissues compared with normal lung tissues both in TCGA dataset and our own cohort. However, the expression of ITGB1-DT was not correlated with prognosis of LUSC in TCGA LUSC dataset. Thus, our findings suggested ITGB1-DT as a LUAD-related lncRNA and implied that ITGB1-DT may be a potential prognostic biomarker for LUAD. Given that many new lncRNAs may not be annotated in the TCGA RNA-seq dataset, we did not exclude the clinical relevance of other lncRNAs in LUAD. The involvements of these lncRNAs in LUAD need further investigation.

To elucidate whether the prognosis-related ITGB1-DT functions as a driver or passenger during LUAD progression, we systematically investigated the potential biological roles of ITGB1-DT in LUAD using *in vitro* cell model and *in vivo* animal model. The gain- and loss-of-function assay results revealed that ITGB1-DT facilitated LUAD cellular proliferation, migration, and invasion *in vitro* and LUAD lung metastasis *in vivo*. Depletion of ITGB1-DT showed the opposite effects. Thus, to our knowledge, these findings first revealed the critical oncogenic roles of ITGB1-DT in LUAD and suggested ITGB1-DT as a potential therapeutic target for LUAD.

Subcellular localization of lncRNAs determines the potential mechanisms of action of lncRNAs. Cytoplasmic lncRNAs may act as microRNA sponges and attenuate the biological roles of interacted microRNAs (Yuan et al., 2014). Cytoplasmic lncRNAs could bind cytoplasmic proteins, while nuclear lncRNAs could bind nuclear proteins (Li et al., 2017; Ma et al., 2020). The bindings between lncRNAs and proteins may influence the stability, localization, and functions of the interacted proteins. Furthermore, cytoplasmic lncRNAs may bind mRNAs and modulate the stability and translation of target mRNAs (Mo et al., 2020). Nuclear lncRNAs may bind pre-mRNAs and regulate pre-mRNA splicing and stability (Xia et al., 2020). Given that *ITGB1-DT* is located on the opposite strand of *ITGB1*, we first investigated the potential regulatory roles of ITGB1-DT on ITGB1. Several lncRNAs have been reported to regulate their nearby genes, such as the transcription promoting roles of NDIME on its nearby gene *MEF2C* and the stabilization of BAZ2B pre-mRNA by lnc-BAZ2B (Bai et al., 2020; Xia et al., 2020). In this study, we identified a positive modulation of *ITGB1* transcription by ITGB1-DT. Bioinformatic prediction and experimental verification demonstrated that ITGB1-DT physically bound to EZH2 and competitively reduced the binding of EZH2 to *ITGB1* promoter. EZH2 is a well-documented epigenetic regulator, which induces H3K27me3 at target gene promoter and represses target gene expression (Karoutas et al., 2019; Karakashev et al., 2020). Consistently, the binding of ITGB1-DT to EZH2 increased ITGB1 expression by reducing the binding of EZH2 to *ITGB1* promoter and H3K27me3 levels at *ITGB1* promoter. The positive regulation of ITGB1 expression by ITGB1-DT was supported by the remarkably positive correlation between the expression of ITGB1 and ITGB1-DT in LUAD tissues. Our findings provided new evidences to support lncRNAs as epigenetic regulators.

ITGB1, also known as integrin β1, has been documented to exert oncogenic roles in various cancers (Wang et al., 2019; Zhang C. et al., 2020). AKT/Wnt/β-catenin pathway is revealed to be a critical downstream mediator of the oncogenic roles of ITGB1 in several cancers (Wang et al., 2019; Zuo et al., 2019). Consistently, in this study, we found that through upregulating ITGB1 expression, ITGB1-DT activated Wnt/β-catenin pathway and further increased Wnt/β-catenin downstream target MYC expression. Intriguingly, ITGB1-DT was also identified as a direct transcriptional target of MYC. As a well-documented oncogenic transcription factor, MYC transcriptionally activated target genes expression. In this study, MYC was also found to transcriptionally activate ITGB1-DT expression. Through activating MYC, Wnt/β-catenin also activated ITGB1-DT expression. In addition, ITGB1 also increased ITGB1-DT expression. Therefore, ITGB1-DT/ITGB1/Wnt/β-catenin/MYC formed a positive feedback loop in LUAD. Functional rescue assays revealed that depletion of ITGB1 or blocking of Wnt/β-catenin pathway reversed the oncogenic roles of ITGB1-DT in LUAD, supporting the ITGB1/Wnt/β-catenin/MYC axis as critical functional mediators of ITGB1-DT.

In summary, our findings identified ITGB1-DT as an upregulated and poor prognosis-related lncRNA in LUAD, which facilitated LUAD progression through activating the ITGB1-DT/ITGB1/Wnt/β-catenin/MYC positive feedback loop (Figure 9E).

## Data Availability Statement

The raw data supporting the conclusions of this article will be made available by the authors, without undue reservation.

## Ethics Statement

The studies involving human participants were reviewed and approved by the Ethics Committee of Xiangya Hospital and were conducted in accordance with the ethical standards of Xiangya Hospital and the Helsinki Declaration. We obtained written informed consents from all participants. The animal experimental procedures were approved by the Animal Ethics Committee of Xiangya Hospital.

## Author Contributions

YG, RC, and XZ designed the study. RC, XX, YF, and XZ performed the experiments. YG, XZ, and CZ supervised the project. YG, RC, and XZ analyzed the data. YG and RC drafted the manuscript. All authors read and approved the final manuscript.

## Conflict of Interest

The authors declare that the research was conducted in the absence of any commercial or financial relationships that could be construed as a potential conflict of interest.

## Footnotes

1 http://gepia.cancer-pku.cn/

2 https://kmplot.com/analysis/

3 http://starbase.sysu.edu.cn/index.php

4 http://lilab.research.bcm.edu/cpat/index.php

5 http://cpc.gao-lab.org/

6 http://genome.ucsc.edu

7 http://pridb.gdcb.iastate.edu/RPISeq/

8 http://jaspar.genereg.net/
