# Supplementary material for: ITGB1-DT Facilitates Lung Adenocarcinoma Progression via Forming a Positive Feedback Loop With ITGB1/Wnt/β-Catenin/MYC
Source: Front Cell Dev Biol. 2021 Mar 4;9:631259. doi: 10.3389/fcell.2021.631259 (PMC7982827; doi:10.3389/fcell.2021.631259)
Supplement: Supplementary file 1 [file Data_Sheet_1.docx]

## Supplementary Figure legends

**Figure 1.** ITGB1-DT induced EMT. **(A)** The expressions of epithelial marker E-cadherin and mesenchymal markers N-cadherin and Vimentin in A549 cells with ITGB1-DT stable overexpression or control were measured by qRT-PCR. **(B)** The expressions of E-cadherin, N-cadherin, and Vimentin in A549 cells with ITGB1-DT stable silencing or control were measured by qRT-PCR. Results are presented as mean ± SD of 3 independent experiments. **P* < 0.05, ***P* < 0.01, ****P* < 0.001 by Student’s *t*-test (A), or one-way ANOVA followed by Dunnett's multiple comparisons test (B).
