# Supplementary figures and images for: ITGB1-DT Facilitates Lung Adenocarcinoma Progression via Forming a Positive Feedback Loop With ITGB1/Wnt/β-Catenin/MYC
Source: Front Cell Dev Biol. 2021 Mar 4;9:631259. doi: 10.3389/fcell.2021.631259 (PMC7982827; doi:10.3389/fcell.2021.631259)

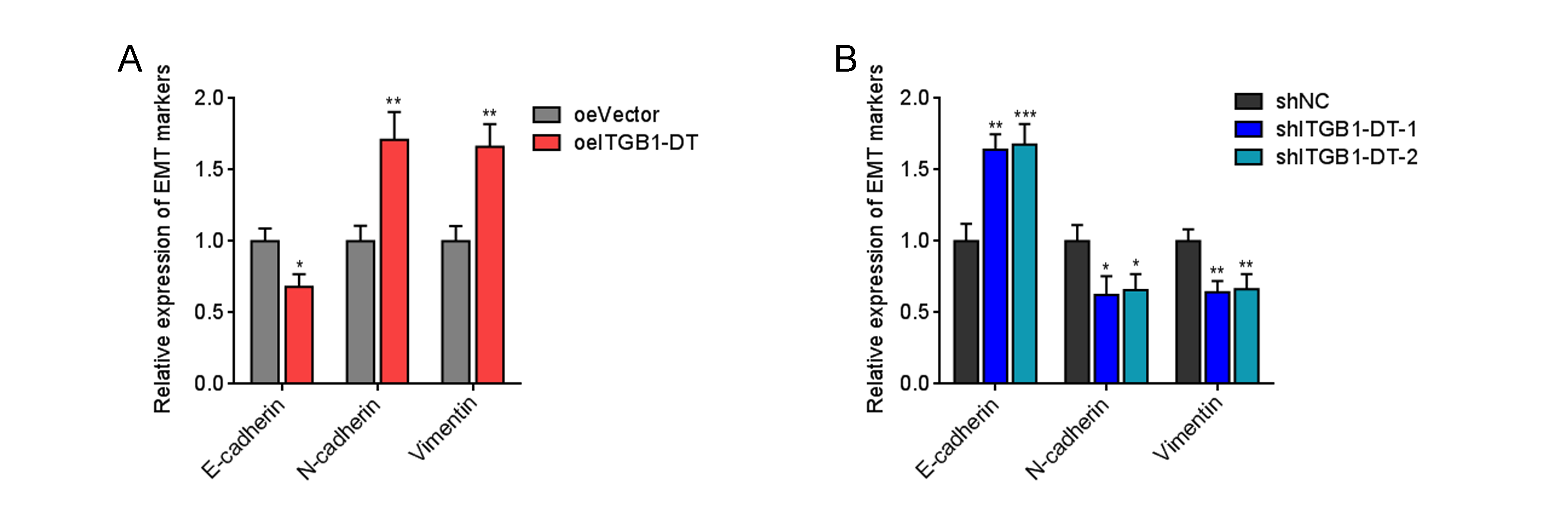

Supplement: Supplementary file 3 [file Image_1.TIF]
